# Developing safe community and healthy city joint model

**DOI:** 10.5249/jivr.v12i3.1343

**Published:** 2020-08

**Authors:** Jafar Sadegh Tabrizi, Homayoun Sadeghi Bazargani, Mohammad Assai Ardakani, Mohammad Saadati

**Affiliations:** ^*a*^ Tabriz Health Services Management Research, Tabriz University of Medical Sciences, Tabriz, Iran.; ^*b*^ Road Traffic Injury Research Center, Health Management and Safety Promotion Research Institute, Tabriz University of Medical Sciences, Tabriz, Iran.; ^*c*^ Ministry of Health and Medical Education,Tehran, Iran.

**Keywords:** Safety, Health promotion, Safe Community, Healthy City

## Abstract

**Background:**

Healthy city and safe community programs are the most common initiatives gaining increasing appeal in various communities to improve safety and health, independently. The aim of this study was to develop a joint application model of safe community and healthy city.

**Methods:**

A comprehensive literature review was conducted on healthy city and safe community programs using PubMed, Web of Science, Scopus and Science Direct and also related websites such as WHO regional offices in 2018. The preliminary list of joint model dimensions and topics were extracted and then assessed by the expert through two rounds of decision Delphi and four expert panel sessions. Eventually, the visual model was developed and approved by the experts.

**Results:**

Literature review resulted in the identification of 11 programs on safety and health promotion in the community of which 35 topics were extracted. After investigating the topics accordance, they were judged (correction, merging or eliminating) by experts through Delphi rounds and panel sessions. Eventually a joint model comprising 14 dimensions, 3 core principles and 4 values called "Safe and Health Promoting Community, SHPC_ model" was developed.

**Conclusions:**

SHPC model provides a parallel and comprehensive view on safety and health topics in a community. The implementation of an integrated model could be one possible way to enhance the commitments on behalf of state and local government, and health system leaders to prioritize injuries and non-communicable disease prevention to address promotion, prevention, treatment and social consequences of mutual community-based interventions.

## Introduction

Nowadays, along with the injuries happening in communities, humans’ health and welfare are also threatened by the various non-communicable diseases. Annually, 5 million people die due to injuries of which 90% is related to developing countries.^1^ Road Traffic Injuries (RTIs), violence, suicide, falls, and unintentional injuries are known to be the common inju-ries worldwide.^2^ A report by transport research laboratory in UK, showed that annual cost of road crashes was about 1% of the GNP in developing countries and 2% in developed countries. The annual global cost of RTIs was estimated to be more than US $ 518 billion.^3^

Alongside, 70% (about 39.5 million) of global mortalities were due to Non-Communicable Diseases (NCDs). Cardiovascular diseases, cancers, diabetes type 2 and chronic lung diseases were among the main NCDs. Evidence showed that through 2011 to 2015 Low-and Middle-Income Countries (LMIC) might lose about 4% of their average GDP annually ($500 billion) due to NCDs morbidity and mortality.^4,5^ Similar to injuries, developing countries has highest shares of NCDs and it is increasing disproportionately.^6-9^ World Health Organization (WHO) mortality and morbidity reports, revealed the growing attention to the prevention of disease and injuries in communities.^7^ As a key aim of public health policies, development of healthy and safe lifestyle in communities was followed by health authorities nationwide and worldwide.^8^

Safe Community movement (SC) and Healthy City program (HC) were two major initiatives encouraged by WHO to promote safety and health in communities^10-12^ SC was introduced in 1989 by Sweden's Karolinska University as a model for community safety promotion and was approved by WHO.^11,12^ As well, HC program was initiated since 1980s in European office of WHO.^13^ These programs’ successful implementation is not dependent on achieving a level of safety and health, but developing cross-sector collaborations, population participation and empowerment, context and evidence-based long term planning were introduced as success criteria.^14-16^

SC and HC programs are implemented independently in the communities. Both are the same in key concepts such as being an inter-sectoral initiative, community-driven, and being based on community evidences. Moreover, there are some complementary items such as bike riding which HC emphasis on to prevent heart disease and SC recommendation of safe biking. A unique view on these programs brings us synergy and efficiency of achieving safety and health promotion goals in the communities. Accordingly, developing a joint model was suggested in a PhD thesis in Iran. The aim of this study was to develop a joint application model of Safe Community & Healthy City programs. 

## Methods 

This research was an applied model development study conducted in Tabriz University of Medical Sciences (TUOMS), Iran, in 2018. A comprehensive literature review was conducted on safe community and healthy city programs using PubMed, Web of Science, Scopus and Science Direct. Moreover, websites of WHO collaborating center on safety promotion in Karolinska Institute and WHO European and Eastern Mediterranean regional offices were reviewed for the relevant documents. Retrieved literature was screened and eligible literature was included. Inclusion criteria were: introducing a model, its dimensions and strategies and providing model description. Included literature were reviewed independently by two researchers and required data on model name, dimensions, topics and values were extracted and then similar items were merged and categorized.

**Preliminary joint application model development:**

First, the accordance of dimensions, topics and values of both extracted SC and HC programs were investigated (Table 1).

**Table 1:** The basic framework and examples for assessing dimensions accordance in SC and HC programs.

Dimensions In two models (examples)	Perfectly matches	Relatively matches	Unique in	
			Healthy city	Safe community
Road Safety		√		
Violence prevention				√
Non-communicable disease management			√	
Risk-groups	√			

This framework was filled independently by two of the research team members who were master in the field. One was expert in public health field with an experience of 25 years and now is one of the health national authorities. The second researcher had 15 years of experience working on safety promotion and injury prevention and is one of the international SC network authorities. In the case of disagreements, the issue was presented in the sessions of the panel of experts. After elaborating on the characteristics of SC and HC programs, a preliminary list of joint application model dimensions and values was developed by the research team. 

**Joint application model assessment**

The preliminary joint application model was assessed via two rounds of Decision Delphi study and 4 expert panel sessions. First, an expert panel session (n= 10) was held and some modifications were done in dimensions and values of preliminary model. Then, the preliminary model was handed over to the experts to be assessed by Delphi questionnaire. 

Delphi questionnaire was designed in a way that the panel members gave scores of 1 to 9 on the model dimensions to the options of dimensions’ applicability in different contexts, the importance of dimensions, and political and cultural acceptance of dimensions in different societies (Table 2). The analysis of results was in a way that the items with median of less than 4 would be eliminated in the first round of Delphi and more than 7 would be considered as the final approved case. Items with a point, from 4 to 7, was sent to the experts to be re-assessed in the second round of Delphi. In addition, necessary modifications in the content of the items was done according to the experts’ opinions. 

**Table 2:** The scoring framework of preliminary model through Delphi study.

Topic:															Dimension:														
Brief Introduction:																													
Comments:																													
Applicability in different contexts										Importance										Political and cultural acceptance in different societies									
To little or no extent					To a very great extent				NA*	To little or no extent					To a very great extent				NA*	To little or no extent					To a very great extent				NA*
1	2	3	4	5	6	7	8	9		1	2	3	4	5	6	7	8	9		1	2	3	4	5	6	7	8	9	

*Not able to answer

After conducting two rounds of Delphi, the results were discussed through two panel sessions (members in each session= 15). Required linguistic modifications in items or categorizing were done based on experts' comments on the joint model. Moreover, some new topics were suggested by the panel members. To review the model values, extracted values were discussed in a dependent panel session and joint model values were finalized. Then, a preliminary visual model was developed by research team and discussed through an independent panel session. After modification, final visual model was developed including joint model dimensions and values. 

The multidisciplinary expert panel consisted of specialists in Health Management and Policy (n=4), Epidemiology (n=3), Health Education and Promotion (n=3), Safety Promotion (n=3), Psychologist (n=1), Primary Care General Practitioners (n=3), Public Health (n=2) and Urban Management (n=2). Inclusion criteria for experts were having scientific experience in the field of safety and health promotion, research experience and participation in the community-based interventions for scientific experts or being experienced in urban management executive, and being experienced in safety areas (traffic, home safety, fire safety, etc.) for at last 5 years.

## Results

Literature review resulted in the identification of 11 programs on safety and health promotion in the community (Appendix 1). After merging similar safety and health promotion topics, presented in various models, their relevancy was reviewed (Table 3).

**Appendix 1:** Retrieved SC and HC programs.

N	Model Name/ Country / year	Domains	Dimensions
1	Cardiff-UK 2010	Reduce inequalities in health and address the differentials in life expectancy across the city	Healthy City
			Homelessness & Housing Need
			Gypsies & Travelers
			Asylum Seekers & Refugees
		Promote healthy lifestyles and prevent ill health	Healthy Weights
			Food & Health
			Physical Activity & Health
			Tobacco Free Cardiff
			Communicable Disease
			Sexual Health
			Substance Misuse
		Improved effectiveness of service delivery to vulnerable adults and children	Mental Health
			Older People
			Learning Disability
			Physical & Sensory Impairment
			Carers
			Chronic Conditions Management
			Domestic & Sexual Violence and Abuse
2	Vancover- Canada A healthy city for all, 2014- 2025	Healthy people(Taking care of the basics)	Expressing ourselves(Enhancing arts, culture and cultural diversity)
			Getting outside (Access to nature)
			Learning for life (Continuous education and development)
			Being active (Opportunities for active living)
			Getting around (Safe, active and accessible transportation)
			A home for everyone (A range of housing options)
			A good start (Healthy childhood development)
			Making ends meet (Adequate income)
			Critical connections (Strong social relationships and support networks)
			Feeding ourselves well (A healthy, just and sustainable food system)
			Being and feeling safe (Addressing fear, violence and crime)
			Human Services (High-quality, accessible and inclusive health, social and community services.)
			Working well (Decent employment conditions)
		Healthy environments (Ensuring livability now and into the future)	A vibrant Social environment
			A thriving economic environment
			A sustainable natural environment
			A well-planned built environment
		Healthy communities (Cultivating connections)	Across the city (Engaged citizenship)
			Out and about (Connecting for belonging at work, at school, at play)
			In the hood (Belonging and inclusion close to home)
3	Tack care New York, 2020	Promote healthy childhood	Baby friendly facilities
			Child care
			Teenage pregnancy
			High school graduation
		Create healthier neighborhoods	Assault hospitalizations
			Fall-related hospitalization
			Air quality
			Homes with no maintenance defects
			Children’s visit to emergency departments for asthma
			Jail population
			Social cohesion
		Support healthy living	Obesity
			Sugary drinks
			Physical activity
			Sodium intake
			Smoking
			Binge drinking
			Overdose death
		Increase access to quality care	Unmet mental health
			Unmet medical need
			Controller high blood pressure
			New HIV diagnosis
			HIV viral suppression
4	Healthy Chicago 2011		Tobacco Use
			Obesity Prevention
			HIV Prevention
			Adolescent Health
			Cancer Disparities
			Heart Disease
			Access to Health Care
			Healthy Mothers
			Communicable Disease
			Healthy Homes
			Violence Prevention
			Public Health
5	Healthy people 2020 America	Physical environment	Environmental Quality
		Social environment	Injury and Violence
			Social Determinants
		Health services	Access to Health Services
			Clinical Preventive Services
			Maternal, Infant, and Child Health
			Mental Health
			Reproductive and Sexual Health
		Individual behavior	Nutrition, Physical Activity, and Obesity
			Oral Health
			Substance Abuse
			Tobacco
			Biology and genetics	--
6	Healthy Chicago 2016-2020		Increasing life expectancy
			Reducing obesity
			Reducing preventable hospitalizations
			Reducing discrimination
			Improving overall health
			Reducing economic hardship
			Increasing opportunities for children to live healthy lives
			Institutionalizing a Health in All Policies approach
			Becoming a Trauma-Informed City
7	Community Safety: A Building Block for Healthy Communities, California: Building Healthy Communities	Improving Places and Systems	Safe public spaces
			Safe schools
			Economic opportunity
			Successful re-entry and re-integration
			Community cohesion
			Community partnerships with criminal justice
		Creating Opportunities for Individual Change	Youth employment
			Transformative mentoring
			Indigenous healing
8	Urban heart		Water, sanitation, food safety, and air pollution
			Health development
			Emergency preparedness and response
			Education and literacy
			Skills development, vocational training, and capacity-building
			Microcredit activities
9	Safe community		Traffic safety
			Occupational safety
			Public Health
			Public places safety
			Home safety
			Violence and suicide prevention
			School safety
			Vulnerable groups safety
			Drugs & Alcohol
			Crime Prevention
			Urban Safety
			Environment – Built & Natural
			Law Enforcement
			Fire & Emergency Services
			Addiction and substance abuse
			Burn prevention
10	European healthy city Phase VI (2014–2018)	The life course and empowering people	Early years
			Older people
			Vulnerability
			Health literacy
		Tackling the major public health challenges in the European Region	Physical activity
			Diet and obesity
			Alcohol
			Tobacco
			Mental well-being
		Strengthening people-centred health systems and public health capacity	Transforming city services delivery
			Revitalizing and strengthening public health capacity
		Creating resilient communities and supportive environments	Community resilience
			Healthy settings
			Healthy urban planning and design
			Healthy transport
			Climate change
			Housing and regeneration
11	Population Health romotion (PHP), Canada	Strengthen Community Action	Income and Social Status
			Social Support Network
		Build Healthy Public Policy	Education
			Working Conditions
		Create Supportive Environments	Physical Environments
		Develop Personal Skills	Biology and Genetics
			Personal Health and Practices and Coping Skills
		Reorient Health Services	Health Child Development
			Health Services

**Table 3:** Safety and Health promotion topics accordance in SC & HC programs.

N	Safety and Health promotion dimensions	Perfectly matches	Relatively matches	Unique in	
				Healthy city	Safe community
1	Traffic Safety		√		
2	Violence prevention		√		
3	Non-communicable disease management			√	
4	Risk-groups health and safety	√			
5	Homes safety				√
6	Leisure times safety				√
7	Children safety	√			
8	Elderly safety				√
9	Work safety		√		
10	Suicide prevention				√
11	Disaster preparedness and response	√			
12	Safe public places		√		
13	Hospitals safety				√
14	Sports safety				√
15	Water safety		√		
16	Schools safety		√		
17	Healthy weight			√	
18	Healthy nutrition			√	
19	Physical Activity promotion			√	
20	Access to nature			√	
21	Tobacco Free		√		
22	Mental well-being		√		
23	Communicable Disease control			√	
24	Addiction and substance abuse prevention		√		
25	Healthy mothers			√	
26	Continuous Skills development			√	
27	Education promotion			√	
28	Physical Environments quality		√		
29	Social Support		√		
30	Home and buildings safety and health	√			
31	Access to Health Services			√	
32	Healthy and safe urban planning and design	√			
33	Air pollution			√	
34	Waste management			√	
35	Sewage system			√	

All the topics presented in Table 3 were assessed and approved by experts in a two-round Delphi study. Items number 20, 26 and 27 were not approved at the first round, but they have got a median score of more than 7 at the second round of Delphi. Approved items were presented in two 1.5-hour expert panel sessions. Experts investigate the approved items and similar items were merged and topics were categorized. Moreover, some topics including life skills development, management of psychiatric disorders, men’s health and safety as a vulnerable group, health literacy promotion, NGOs development and violence surveillance were suggested by panel members and added to the topics. Retrieved model values were discussed in a separate panel session and finally seven values (3 as core principles and 4 as ruling) were approved. 

**Figure 1:**
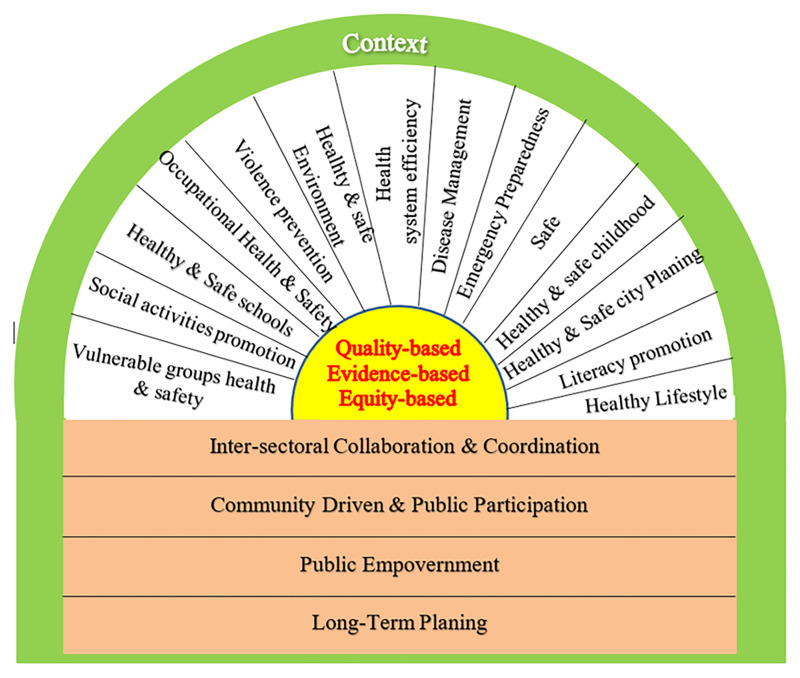
Safe & Health Promoting Community Model.

Finally, after modification of preliminary visual model by experts, the joint model called "Safe and Health Promoting Community (SHPC) model" was developed comprising 7 values and 14 main dimensions to improve safety and health in the community (Figure 1). Each dimension included especial topics being presented in Table 4.

**Table 4:** SHPC dimensions and topics.

N	Dimension	Topics
1		Safe behaviour
		Healthy Nutrition
	Healthy lifestyle	Physical activity
		Tobacco free community
		Life skills development
2		Non-communicable disease management
	Disease management	Communicable Disease control
		Management of psychiatric disorders
3		Addiction and substance abuse prevention
		Elderly Health & Safety
	Vulnerable groups Health & Safety	Disabled people
		Men Health & Safety
		Pregnant women
4		Education promotion
	Literacy promotion	Health Literacy promotion
		Continuous Local Skills development
5		Healthy water
		Clean air
	Healthy & safe Environment	Healthy food
		Safe waste management
		Access to sewage system
6		Safe traffic culture
	Safe traffic	Safe traffic environment
		Safe vehicle
7	Healthy & safe childhood	Healthy childhood services promotion
8		Social groups support
	Social activities promotion	NGOs development
		Social networks promotion
9		Access to health services
	Health system efficiency	health services utilization
		health services quality
10		Safe urban furniture
	Healthy & Safe urban Planning	Safe public places
		Safe leisure places
		Safe home
11	Violence prevention	Violence Surveillance
		Community-based Violence prevention
12	Emergency Preparedness	Crisis management promotion
		Community preparedness
13	Healthy & Safe schools	
14	Occupational Health & Safety	

## Discussion

This study contributed in developing a joint application model of "Safe Community" and "Healthy City" programs called "Safe and Health Promoting Community (SHPC) Model (Sahand Model)". Creating a comprehensive and parallel view on community safety and health promotion, forming an infrastructure for inter-sectoral collaboration, and public participation are the main potential advantages of the SHPC model. Moreover, it encourages directing the community resources through targeted strategies and turns safety and health to be a political choice for city administrates and contributes in more efficiency and effectiveness of safety and health promotion initiatives in the communities.^17^ Parallel and comprehensive attention on safety and health issues is the distinguished and different characteristic of SHPC when compared to common models. For instance, the Vancouver "Healthy City for All (2014-2025)" strategy, beside the health issues, includes only two safety topic of crime prevention and safe built environment.^18^ Also, in the HC program of WHO Eastern Mediterranean Regional called "Urban Heart", major focus was on the health topics such as maternal health, environmental health and so on.^19^ The SHPC model dimensions and topics, covers the most common health and safety issues in a community and also brings the different models components under an umbrella which result in an effective policy making for safety and health promotion in local level. In another word, the SHPC model have the potential to eliminate the policy makers concerns on thinking about various models of safety and health promotion programs to implement in their communities.

Literature revealed that safety is a prerequisite for health.^12^ Thus, the parallel attention on safety and health in SHPC, especially in interacted issues, such as biking which healthy city program focus on physical activity promotion through biking, but SHPC suggests safe biking using safety equipment, will lead to synergy in achievement of defined vision.

Public empowerment and participation are concepts that are highly focused in new theories of safety and health promotion.^11,17,20^ Now, is the issue has changed as a political connotation and must be a part of main strategy of community’s political body. SHPC model focuses on public empowerment and participation issues as model values that are umbrella terms for all the safety and health promotion initiatives. Pervious literature such as safe community manifesto (1989) and Ottawa charter (1986), have announced equity as a principle in community safety and health promotion.^20,21^ Core values of SHPC including quality, evidence and equity-based safety and health promotion provides three main decision-making principles of model execution in communities. It means that, all policies and initiatives in SHPC projects must be developed based on evidences and considering equity promotion in the community. Moreover, to improve the initiatives effectiveness, action plans must be developed considering qualitative measures.

It was stated that safety and health promotion initiatives should enable, advocate and mediate. Considering the SHPC model values and principles, it has the potential to successfully advocate through collaborative structure, creating equal opportunities for people enabling them to achieve an acceptable level of health and bring the commitment of policy-makers to create a safe and health promoting community.

## Conclusion

Key to the success of integrated “Safe and Health Promoting Community” model is its potential to integrate and combine the capabilities of community, public and private sectors, NGOs and local political and social authorities to create a local community-based movement able to influence the policy agenda in state level and action plan in local level. The implementation of this integrated model could be one possible way to strengthen the commitments paid by state and local government and health system executives to priorities injuries and NCDs prevention to address promotion, prevention, treatment and social consequences of mutual community-based interventions. 

**Limitation:**

Feasibility is one of the most important characteristics of community-based models. As the feasibility study of the SHPC will take longer time, it will be reported in future studies. Moreover, the joint model was developed based on international requirements of the safe community and healthy city programs with Iran characteristics consideration. It may need some minor adaptations when using in other settings. 

**Acknowledgment**

We are thankful of all the experts who participated in the study.

## References

[1] World Health Organization. Injuries and Violence: The Facts. World Health Organization. Geneva; 2014.

[2] Bachani AM, Zhang XJ, Allen KA, Hyder AA (2014). Injuries and violence in the Eastern Mediterranean Region: a review of the health, economic and social burden. East Mediterr Health J.

[3] Jacobs G, Aeron-Thomas A, Astrop A. Estimating global road fatalities. Crowthorne, United KingdomTransport Research Laboratory (TLR Report445). 2000, http://citeseerx.ist.psu.edu/viewdoc/download?doi=10.1.1.174.5207&rep=rep1&type=pdf, accessed 07 September 2018.

[4] Bloom D, Chisholm D, Jan-Llopis E, Prettner K, Stein A, Feigl A, et al. From burden to" best buys": reducing the economic impact of non-communicable disease in low-andmiddle-income countries. Program on the Global Demography of Aging; 2011, https://www.who.int/nmh/publications/best_buys_summary/en/, accessed 07 August 2018.

[5] Ding D, Lawson KD, Kolbe-Alexander TL, Finkelstein EA, Katzmarzyk PT, van Mechelen W (2016). The economic burden of physical inactivity: a global analysis of major non-communicable diseases. Lancet.

[6] World Health Organization. World health statistics 2015: World Health Organization; 2015, https://www.who.int/gho/publications/world_health_statistics/2015/en/, accessed 5 August 2018.

[7] World Health Organization. NCD mortality and morbidity: world Health Organization, 2016, https://www.who.int/gho/ncd/mortality_morbidity/en/,accessed 5 August 2018.

[8] Cummins S, Ogilvie D, White M, Petticrew M, Jones A, Goodwin D, et al. National Evaluation of the Healthy Communities Challenge Fund: The Healthy Towns Programme in England,Technical Report, 2016, https://researchonline.lshtm.ac.uk/id/eprint/3163750, accessed 1 August 2018.

[9] Hosseinpoor AR, Bergen N, Mendis S, Harper S, Verdes E, Kunst A (2012). Socioeconomic inequality in the prevalence of noncommunicable diseases in low-and middle-income countries: results from the World Health Survey. BMC Public Health.

[10] Leeuw ED (2009). Evidence for healthy cities: reflections on practice, method and theory. Health Promot Int.

[11] Tabrizi JS, Sadeghi-Bazargani H, Mohammadi R, Saadati M (2018). Iranian designated Safe Communities: a quantitative analysis. Trauma Monthly.

[12] Welander G, Svanstrm L, Ekman R (2004). safety promotion-an introduction. Stockholm: Karolinska Institutet.

[13] Janss Lafond L, Heritage Z (2009). National networks of healthy cities in Europe. Health Promotion International.

[14] Plümer KD, Kennedy L, Trojan A (2010). Evaluating the implementation of the WHO Healthy Cities Programme across Germany (1999–2002).. Health Promotion International.

[15] Wang S, Zou J, Yin M, Yuan D, Dalal K (2011). Injury epidemiology in a safe community health service center in Shanghai, China. Health MED.

[16] Dooris M, Heritage Z (2013). Healthy Cities: facilitating the active participation and empowerment of local people. J Urban Health.

[17] De Leeuw E, Simos J. Healthy Cities: The theory, policy, and practice of value-based urban planning: Springer, 2017:26-50.

[18] Social Policy Division. A Healthy City for All; Vancouvers Healthy City Strategy 2014-2025 | Phase 1. City of Vancouver, Community Services, 2014, https://vancouver.ca/people-programs/healthy-city-strategy.aspx, accessed 05 June 2019.

[19] World Health Organization. Urban HEART: urban health equity assessment and response tool: user manual. World Health Organization, Geneva, 2010: 22-30.

[20] Ottawa charter for health promotion, Health Promotion International, Volume 1, Issue 4, 1986, Page 405, https://doi.org/10.1093/heapro/1.4.405.

[21] Ottawa charter for health promotion, Health Promotion International. 1986, https://apps.who.int/iris/bitstream/handle/10665/79061/9789241500784_eng.pdf, accessed 10 August 2018.

